# Beliefs about the Harmfulness of Heated Tobacco Products Compared with Combustible Cigarettes and Their Effectiveness for Smoking Cessation among Korean Adults

**DOI:** 10.3390/ijerph17155591

**Published:** 2020-08-03

**Authors:** Seung Hee Kim, Seo Young Kang, Hong-Jun Cho

**Affiliations:** 1 Department of Family Medicine, Asan Medical Center, University of Ulsan College of Medicine, Seoul 05505, Korea; kohakush@naver.com; 2 International Healthcare Center, Asan Medical Center, Seoul 05505, Korea; sykang@amc.seoul.kr

**Keywords:** heated tobacco products, cigarette smoking, risk, perception, smoking cessation

## Abstract

Heated tobacco products (HTPs) have been widely used in Korea since their introduction in 2017. In this study, we investigated the perceptions of their relative harmfulness and smoking cessation effects. We performed an online survey in 7000 Koreans in 2018 (2300 males and 4700 females aged 20–69 years) by matching their age, sex, and provincial distribution. To investigate the factors causing HTPs to be perceived as less harmful than combustible cigarettes (CCs) and helpful for smoking cessation, we used multivariable logistic regression analyses. HTPs were less harmful than CCs in 16.8% of participants, particularly among HTP-only users and dual and triple users of HTPs, electronic cigarettes (ECs), or CCs than among CC-only users, those who were aged ≤ 34 years, males, and those with higher incomes. HTPs were reportedly helpful for smoking cessation in 11.2% of participants. Similar perceptions were more likely among HTP-only users, as well as dual and triple users than among CC-only users and adults with higher education/incomes. Although Korean adults generally had negative perceptions of the harmfulness and smoking cessation effects of HTPs compared with CCs, dual and triple users were more likely to have positive perceptions. Monitoring the use of multiple tobacco products and HTPs is a new challenge for Korean policymakers.

## 1. Introduction

Heated tobacco products (HTPs) comprise small cigarettes, in which the tobacco is heated just enough to release a flavorful nicotine-containing vapor [1]. On the other hand, electronic cigarettes (ECs) are battery-powered devices that vaporize a liquid solution containing nicotine and flavorants [2]. HTPs electrically heat the tobacco without burning to a lower temperature than combustible cigarettes (CCs) to produce an aerosol with nicotine. This is unlike ECs, in which users inhale the aerosol created by heating a nicotine liquid [3]. One brand of HTPs was introduced to South Korea (hereafter, Korea) in June of 2017. The market share of HTPs rapidly increased and reached 10.5% of Korea’s total sales of tobacco products in 2019 [4]. Among Korean men, the prevalence of CC use was 61.8% in 2001, but by 2018, it decreased to 36.7%; whereas among Korean women, its prevalence increased from 5.4% in 2001 to 7.5% in 2018 [5]; which was still very low compared with that of Korean men.

Previous studies have been conducted on the correlates of HTP use among Korean adults [6,7]. Demographic and socioeconomic characteristics associated with using HTPs include being male, young, and having a high education level and economic status. HTPs are marketed as clean, sophisticated, high-tech products that provide all of the benefits of smoking with less ash and odor [8]. These characteristics could attract young, male adults. Due to the high maintenance and cost of using HTP devices, people with a higher socioeconomic level were more likely to use them. Moreover, the use of CCs or ECs was the most significant factor related to the use of HTPs [6,7], which may be because CC or EC users were more likely to have a positive perception of HTPs due to marketing claims that indicate they are less harmful than CCs [8].

Beliefs and perceptions about the health risks associated with smoking play significant roles in reducing its prevalence, as concerns about such risks have been reported as the most common motivation to quit smoking and prevent smoking initiation [9,10]. With regard to the association between the perceptions of relative harmfulness of HTPs and their usage, as compared to non-HTP users, HTP users were more likely to believe HTPs to be less harmful than CCs, with this belief being more prominent among its frequent users [11]. Furthermore, despite understanding that HTPs were not risk-free, most reported that they used HTPs due to their beliefs about its having health benefits over CCs [6], with one of the main reasons being their perception of its reduced harm [12]. These results suggest that the perception of HTPs being less harmful than CCs may have had a major impact on HTP usage in Korea.

There is controversy regarding whether HTPs are less harmful than CCs; despite HTPs being marketed with claims of their being less harmful based on assertions that they expose users to lower levels of toxicants [13]. While the levels of some harmful and potentially harmful constituents of HTPs were lower than those of CCs [14], the reduced exposure of some toxicants does not mean a reduced risk of harm to health [13]. Since the health risks of HTPs have not been determined, further studies are needed.

Even though one of the reasons for using HTPs was to stop smoking [6,12], several studies have shown no significant association between the use of HTPs and smoking cessation-related behaviors, including attempts or intentions to quit smoking [7,15,16]. Moreover, adolescent HTP users were less likely to become former CC smokers despite multiple attempts at smoking cessation [17]. This was probably because although some smokers initiated HTPs for smoking cessation, CC users were more likely to become dual users of HTPs and CCs rather than succeeding in quitting smoking. Regarding perceived effects of HTPs on smoking cessation; a recent study showed that users rather than non-users of ECs were more likely to believe that HTPs are helpful in quitting smoking [18].

Despite the current lack of evidence regarding the long-term health effects of HTPs and their effectiveness for smoking cessation, marketing claims of HTPs’ reduced health risks could have misled consumers [13]. To our knowledge, no research has assessed the perceptions of HTPs in a large population of Korean adults. Therefore, this study aimed to investigate the beliefs about the relative harmfulness of HTPs and CCs, as well as their effectiveness for smoking cessation among Korean adults. 

## 2. Materials and Methods

### 2.1. Study Sample

We conducted an online survey using a panel managed by the research company EMBRAIN (Seoul, Korea), with 1.3 million members (as on December 2018). We randomly invited 70,000 individuals by matching their age, sex, and provincial distributions with the 2018 national population statistics. However, only 10,489 responded, from which we included 7000 (2300 men and 4700 women aged 20–69 years), after excluding 2285 who outnumbered our quota, 21 who did not meet the age criteria, 400 who decided not to participate in the survey, and 783 who responded incorrectly. We oversampled twice as many women as men because the prevalence of tobacco product use was much lower among Korean women than men.

Data were collected from 3–9 November 2018. All participants received financial incentives equivalent to 5000 Korean Republic Won (KRW), equal to approximately 5 US Dollars (USD). All the participants gave their informed consent for inclusion, prior to participating in the study. The study was conducted in accordance with the Declaration of Helsinki, and its protocol was approved by the Asan Medical Center Institutional Review Board (S2018-1662-0001) in Seoul, Korea. 

### 2.2. Measures

#### 2.2.1. Status of Tobacco Product Use and Beliefs about the Relative Harmfulness and Smoking Cessation Effects of HTPs

Current CC users were defined as adults who had smoked at least 100 cigarettes during their lifetime and reported currently smoking “every day” or on “some days”. Former CC users were defined as adults who had smoked at least 100 cigarettes but had responded “not at all” to the question about current smoking. Never-CC users were defined as adults who had neither smoked at least 100 cigarettes nor ever smoked CCs in their lifetime. Current HTP users were defined as adults who had continually used HTPs in their lifetime and reported currently using them “every day” or on “some days”. Former HTP users were defined as adults who had frequently used HTPs in their lifetime but were currently not using them. Never-HTP users were defined as adults who had never used HTPs in their lifetime. Current EC users were defined as adults who had repeatedly used ECs in their lifetime, as well as during the past month. Former EC users were defined as adults who had habitually used ECs in their lifetime but had not used them during the past month. Never-EC users were defined as adults who had never used ECs in their lifetime. Since the prevalence of using other tobacco products (e.g., snus, water pipes, cigars, chewing tobacco, pipes, rolling tobacco, snuff) was negligible (less than 0.01%) among Korean adults [7], we did not include questions on these in our survey.

The status of tobacco product use was classified into nine groups. “Never-users of any of the three tobacco products” were defined as adults who had never used CCs, HTPs, or ECs. “Former users of any of the three tobacco products” included former CC, HTP, and EC users who were currently not using any of the three products. “Current users of any of the three tobacco products” included “CC-only users”, “HTP-only users”, “EC-only users”, “dual users of HTPs and CCs”, “dual users of ECs and CCs”, “dual users of HTPs and ECs”, and “triple users of the three products”.

Perceptions of the relative harmfulness of HTPs and CCs was evaluated with the question: “What do you think is the effect of ‘heated tobacco products’ on your health as compared with CCs?” The response options were: “They are less harmful”, “They are similar”, and “They are more harmful”. Whether HTPs can help with smoking cessation was evaluated with the question: “Do you think ‘heated tobacco products’ are helpful for quitting smoking?” The response options were: “Yes”, “No”, and “I am not sure”.

#### 2.2.2. Sociodemographic Characteristics

The sociodemographic characteristics analyzed included age, sex, education, monthly household income, and marital status.

### 2.3. Data Analysis

We calculated the proportion of participants in each category of sociodemographic characteristics and the prevalence of tobacco product use. Furthermore, we evaluated the proportion of participants believing in the health benefits of HTPs being relative to CCs, and those perceiving HTPs as effective for smoking cessation. To identify factors associated with the perceptions of HTPs, adjusted odds ratios (AOR) and 95% confidence intervals (CI) were obtained via multivariable logistic regression analysis, after adjusting for potential confounders. Data analyses were conducted using IBM SPSS Statistics for Windows, Version 24.0 (IBM Corp., Armonk, NY, USA).

## 3. Results

Around 30% of the participants were aged 20–34 years, while those aged 35–49 and 50–69 years were each around 35%. Approximately 60% had college-level education, and over 50% had monthly household incomes of 3–6.99 million KRW. The overall prevalence of current use of tobacco products, including CCs, HTPs, and ECs was 27.4% (44.1% among men and 11.0% among women) (Table 1).

**Figure 1:**
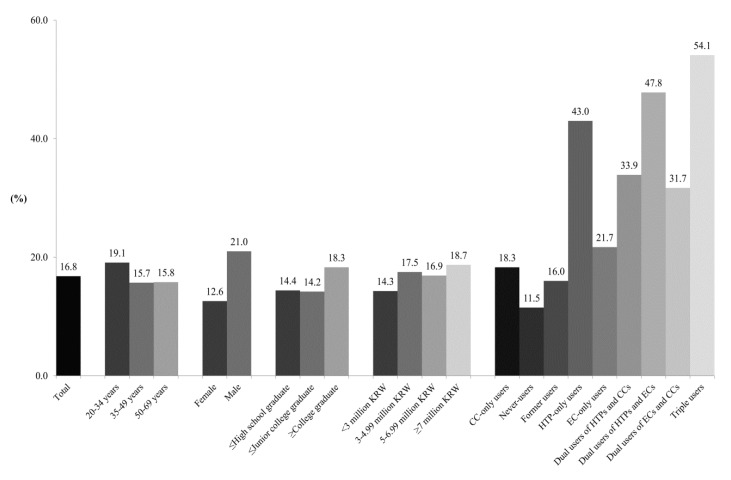
Frequency of the belief that HTPs are less harmful than CCs. Abbreviations: CCs = combustible cigarettes; HTPs = heated tobacco products; ECs = electronic cigarettes; KRW = Korean Republic Won (KRW 1000 = US Dollar 1). Values are presented as weighted percentages (the weighted % of women was 50).

Of the 7000 participants, 16.8% reported HTPs to be less harmful than CCs (Figure 1). Using multivariable logistic regression analysis (Table 2), participants aged ≤ 34 years (AOR = 1.20, 95% CI = 1.00, 1.44), who were male (AOR = 1.26, 95% CI = 1.11, 1.44), and had an income ≥7 million KRW (AOR = 1.30, 95% CI = 1.08, 1.57) were associated with greater odds of perceiving HTPs as less harmful than CCs, as compared with those who were aged ≥ 50 years, female, and had an income <3 million KRW, respectively. As compared with CC-only users, never-users of any of the three tobacco products had lower odds of perceiving HTPs as less harmful than CCs (AOR = 0.64, 95% CI = 0.54, 0.77). However, HTP-only users (AOR = 3.32, 95% CI = 2.25, 4.90), dual users of HTPs and CCs (AOR = 2.25, 95% CI = 1.77, 2.86), dual users of HTPs and ECs (AOR = 3.96, 95% CI = 2.17, 7.24), dual users of ECs and CCs (AOR = 1.96, 95% CI = 1.39, 2.77), and triple users (AOR = 5.12, 95% CI = 3.93, 6.67) had greater odds of perceiving HTPs as less harmful than CCs.

**Figure 2:**
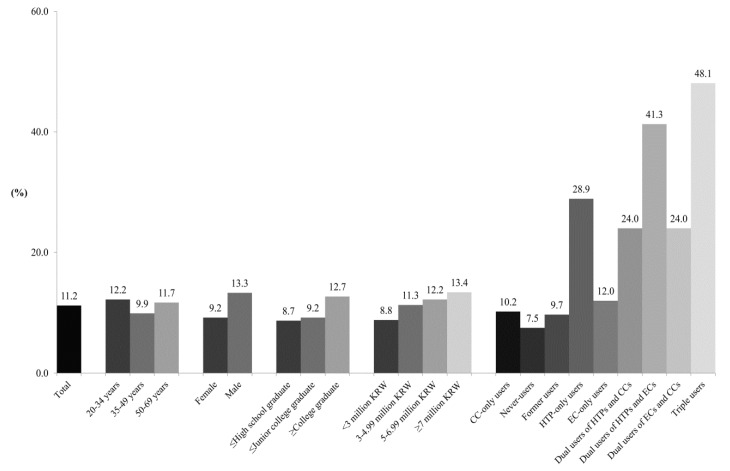
Frequency of the belief that HTPs aid in smoking cessation. Abbreviations: CCs = combustible cigarettes; HTPs = heated tobacco products; ECs = electronic cigarettes; KRW = Korean Republic Won (KRW 1000 = US Dollar 1). Values are presented as weighted percentages (the weighted % of women was 50).

As for the perceived effectiveness of HTPs for smoking cessation, 11.2% of participants reported HTPs as effective for smoking cessation (Figure 2). Based on multivariable logistic regression analysis (Table 2), groups with education ≥ college graduate (AOR = 1.35, 95% CI = 1.12, 1.63) and income ≥7 million KRW (AOR = 1.43, 95% CI = 1.15, 1.78) were more likely to agree with the effectiveness of HTPs for smoking cessation. As compared with CC-only users, never-users of any of the three tobacco products had lower odds of perceiving HTPs as effective for smoking cessation (AOR = 0.72, 95% CI = 0.57, 0.90). However, HTP-only users (AOR = 3.61, 95% CI = 2.34, 5.59), dual users of HTPs and CCs (AOR = 2.84, 95% CI = 2.14, 3.75), dual users of HTPs and ECs (AOR = 6.01, 95% CI = 3.23, 11.19), dual users of ECs and CCs (AOR = 2.81, 95% CI = 1.90, 4.14), and triple users (AOR = 7.93, 95% CI = 5.94, 10.57) had greater odds of believing HTPs to be effective for smoking cessation.

## 4. Discussion

Overall, participants were less likely to believe in the relative health benefits of HTPs and CCs. Only 16.8% of the participants believed that HTPs were less harmful than CCs, which was lower than the results of a Canadian study, in which 25% of the participants perceived HTPs as less harmful than CCs [18]. The negative public perceptions of HTPs are understandable, considering they contain real tobacco and there was widespread media coverage when the Korea Ministry of Food and Drug Safety released information on harmful HTP emissions in 2018 [19]. These negative perceptions of HTPs can also be ascribed to Korea’s relatively stringent tobacco regulations. While Korean tobacco control policies—which include smoking bans in public places, regulations on advertising, and bans on sales to minors—are applied equally to CCs and HTPs, the individual consumption tax for HTPs is 89% of the tax levied on CCs [20]. 

The perception of the relative harmfulness of HTPs compared with CCs differed according to the pattern of tobacco product use. Compared with CC-only users, HTP users were more likely to perceive HTPs to be less harmful than CCs. Consistent with these results, a previous study found that HTP-only users and dual users of HTPs and CCs were more likely to believe that HTPs were less harmful to users than CCs, compared with CC-only users [16]. It is common for some tobacco product users to choose specific products which they perceive as less harmful because of their favorable perceptions towards these products [21,22,23,24]. Moreover, the odds of perceiving HTPs as less harmful than CCs were highest among triple users of the three products. Since adults with no perception of risk regarding HTPs or ECs were more likely to use multiple tobacco products than those who perceived a risk [25], triple users would have been less likely to have risk perceptions of HTPs.

Intriguingly, both HTP and EC users perceived HTPs to be less harmful than CCs. EC users’ perceptions of the relative harmfulness of HTPs were consistent with the results of a previous study which reported that EC users, rather than non-EC users were more likely to believe that HTPs were less harmful than CCs [18]. An explanation could be that EC users considered HTPs to be similar because of the similarity of the Korean terms for ECs and HTPs, whose direct translations were “liquid-type electronic cigarettes” and “cigarette-type electronic cigarettes”, respectively. This could have made people regard the two products as similar and caused confusion in distinguishing them. Furthermore, HTPs and ECs have many similarities in that they are marketed as being less harmful than CCs and as being clean and emitting less odor [8,26]. 

As for the perceived effects of smoking cessation of HTPs, 11.2% of participants believed HTPs to be effective for smoking cessation, which was much lower than the results of a Canadian study which reported that 35% of participants considered HTPs effective for smoking cessation [18]. According to previous Korean studies, about 30% of HTP users agreed that HTPs helped people quit CC smoking, which was much lower than the 79% and 76% of participants who believed the benefits of HTPs to be lack of ash and less odor, respectively [27], and cited its being less harmful and having less smelly characteristics as their major reasons for using HTPs [6]. Furthermore, in a Korean qualitative interview study, most current or former HTP users did not believe that HTPs had a positive effect on contemplation of smoking cessation [28]. Therefore, positive perceptions on the effects of HTPs on smoking cessation were not the main driver, though they could have contributed to the use of HTPs in Korea. It is intriguing that triple users highly rated HTPs as effective for smoking cessation. Perhaps they were working toward quitting, but they became poly-tobacco users instead of quitting smoking.

The popularity of HTPs in Korea, despite the generally negative perceptions on its health benefits compared with CCs and smoking cessation effects, can be attributed to successful marketing by tobacco industries and consumers being attracted to its other characteristics (e.g., less odor, no ash, fancy appearance) [8,26]. Among smokers, CC users who had been exposed to HTP advertising were more likely to perceive HTPs as less harmful than CCs [11]. The advertisements on reduced exposure claims of HTPs may have resulted in smokers having a positive perception of HTPs and using them [13]. Previous research indicates that EC users were more likely to have positive perceptions about HTPs and may be more susceptible to the marketing of these products [7], which may lead to HTP use. Presumably, various factors may have interacted with one another and influenced the popularity of HTPs [12]. 

Our study had a few limitations. The prevalence of tobacco product use needs to be interpreted cautiously, since our sample was not nationally representative; however, we tried to match sex, age, and provincial distributions in line with national statistics. As this study was cross-sectional, causal relationships between the perceptions of HTPs and their use could not be identified. The participants may not have completely distinguished HTPs from ECs due to their Korean terms being similar, and also because HTPs were new products at the time of data collection. Moreover, a standardized questionnaire for the use of HTPs was not available during the study period. To mitigate these problems in the questionnaire, we included pictures and names of HTPs available in the Korean market and presented the questions on HTPs after those on ECs [29]. A longitudinal study is needed to investigate the causal relationships between perceptions of HTPs and their use, including a nationally representative sample and a standardized questionnaire on HTP use.

## 5. Conclusions

Perceptions about the relative harmfulness and smoking cessation effects of HTPs were generally negative among Korean adults. However, dual and triple users of HTPs, ECs, or CCs were more likely to have positive perceptions about HTPs, which may have led to their use or otherwise lead people to use them. Therefore, more emphasis on monitoring the use of multiple tobacco products and HTPs is needed to cope with the increasing popularity of HTPs in Korea.

## Figures and Tables

**Table 1:** Basic characteristics of the study participants (*n* = 7000).

Characteristic	Men (*n* = 2300)		Women (*n* = 4700)		Total
	*n* ^a^	% ^a^	*n*	%	%
**Age (years)**					
20–34	690	30.0	1437	30.6	30.3
35–49	833	36.2	1612	34.3	35.2
50–69	777	33.8	1651	35.1	34.5
**Education**					
≤High school graduate	416	18.1	1129	24.0	21.1
≤Junior college graduate	336	14.6	1005	21.4	18.0
≥College graduate	1548	67.3	2566	54.6	60.9
**Monthly household income (KRW 1000) ^b^**					
<3000	618	26.9	1156	24.6	25.7
3000–4999	817	35.5	1614	34.3	34.9
5000–6999	476	20.7	1078	22.9	21.8
≥7000	389	16.9	852	18.1	17.5
**Marital status**					
Never married	823	35.8	1567	33.3	34.5
Married/cohabited	1399	60.8	2910	61.9	61.4
Separated/divorced/bereaved	78	3.4	223	4.7	4.1
**Tobacco product use status**					
Never-users of any of the three tobacco products	760	33.0	3906	83.1	58.3
Former users of any of the three tobacco products	527	22.9	277	5.9	14.3
Current users of any of the three tobacco products	1013	44.1	517	11.0	27.4
CC-only users	541	23.5	248	5.3	14.3
HTP-only users	44	1.9	33	0.7	1.3
EC-only users	27	1.2	29	0.6	0.9
Dual users of HTPs and CCs	196	8.5	74	1.6	5.0
Dual users of HTPs and ECs	13	0.6	20	0.4	0.5
Dual users of ECs and CCs	72	3.1	39	0.8	2.0
Triple users	120	5.2	74	1.6	3.4

Abbreviations: CCs = combustible cigarettes; HTPs = heated tobacco products; ECs = electronic cigarettes. ^a^ Values are presented as unweighted numbers and weighted percentages (the weighted % of women was 50). ^b^ Korean Republic Won (KRW) is Korea’s currency (KRW 1000 = US Dollar 1).

**Table 2:** Comparison of beliefs about the harmfulness of HTPs compared with CCs, and the perceived effectiveness of HTPs for smoking cessation (*n* = 7000).

Factor	HTPs are Less Harmful than CCs		HTPs are Helpful for Smoking Cessation	
	Crude	Multi-Adjusted ^a^	Crude	Multi-Adjusted ^a^
	OR (95% CI)	AOR (95% CI)	OR (95% CI)	AOR (95% CI)
**Age (years)**				
50–69	1	1	1	1
35–49	0.99 (0.87, 1.13)	0.90 (0.78, 1.04)	**0.83 (0.71, 0.97)**	**0.72 (0.61, 0.86)**
20–34	**1.25 (1.10, 1.43)**	**1.20 (1.00, 1.44)**	1.05 (0.90, 1.22)	0.94 (0.76, 1.17)
**Sex**				
Female	1	1	1	1
Male	**1.85 (1.65, 2.06)**	**1.26 (1.11, 1.44)**	**1.52 (1.33, 1.73)**	0.98 (0.84, 1.15)
**Education**				
≤ High school graduate	1	1	1	1
≤ Junior college graduate	0.99 (0.82, 1.19)	0.97 (0.80, 1.18)	1.07 (0.85, 1.34)	1.09 (0.86, 1.38)
≥ College graduate	**1.34 (1.16, 1.54)**	1.15 (0.99, 1.34)	**1.52 (1.27, 1.81)**	**1.35 (1.12, 1.63)**
**Monthly household income (KRW 1000) ^b^**				
<3000	1	1	1	1
3000–4999	**1.27 (1.09, 1.47)**	**1.27 (1.09, 1.48)**	**1.33 (1.11, 1.59)**	**1.29 (1.07, 1.56)**
5000–6999	**1.22 (1.03, 1.43)**	1.17 (0.98, 1.41)	**1.44 (1.19, 1.75)**	**1.31 (1.05, 1.62)**
≥7000	**1.37 (1.16, 1.62)**	**1.30 (1.08, 1.57)**	**1.60 (1.31, 1.96)**	**1.43 (1.15, 1.78)**
**Marital status**				
Never married	1	1	1	1
Married/cohabited	0.92 (0.82, 1.03)	0.97 (0.83, 1.14)	1.01 (0.88, 1.15)	0.95 (0.78, 1.15)
Separated/divorced/bereaved	0.85 (0.63, 1.13)	0.98 (0.71, 1.37)	1.02 (0.73, 1.42)	1.09 (0.75, 1.59)
**Tobacco product use status**				
CC-only users	1	1	1	1
Never-users of any of the three tobacco products	**0.58 (0.49, 0.68)**	**0.64 (0.54, 0.77)**	**0.72 (0.59, 0.88)**	**0.72 (0.57, 0.90)**
Former users of any of the three tobacco products	0.85 (0.70, 1.04)	0.85 (0.69, 1.04)	0.95 (0.74, 1.23)	0.92 (0.71, 1.18)
HTP-only users	**3.37 (2.29, 4.96)**	**3.32 (2.25, 4.90)**	**3.60 (2.34, 5.55)**	**3.61 (2.34, 5.59)**
EC-only users	1.24 (0.72, 2.13)	1.18 (0.68, 2.03)	1.21 (0.61, 2.40)	1.13 (0.57, 2.24)
Dual users of HTPs and CCs	**2.30 (1.81, 2.91)**	**2.25 (1.77, 2.86)**	**2.80 (2.12, 3.70)**	**2.84 (2.14, 3.75)**
Dual users of HTPs and ECs	**4.10 (2.26, 7.43)**	**3.96 (2.17, 7.24)**	**6.23 (3.37, 11.50)**	**6.01 (3.23, 11.19)**
Dual users of ECs and CCs	**2.08 (1.48, 2.92)**	**1.96 (1.39, 2.77)**	**2.80 (1.91, 4.11)**	**2.81 (1.90, 4.14)**
Triple users	**5.28 (4.06, 6.86)**	**5.12 (3.93, 6.67)**	**8.20 (6.17, 10.89)**	**7.93 (5.94, 10.57)**

Abbreviations: HTPs = heated tobacco products; CCs = combustible cigarettes; ECs = electronic cigarettes; OR = odds ratio; AOR = adjusted odds ratio; CI = confidence interval. ^a^ Multiple logistic regression analysis adjusted for age, sex, education, income, marital status, and tobacco products use status. ^b^ Korean Republic Won (KRW) is Korea’s currency (KRW 1000 = USD 1). Bold values were significantly associated (*p* < 0.05).
